# Adding of Sitagliptin on Insulin Therapy Effectively and Safely Reduces a Hemoglobin A1c Level and Glucose Fluctuation in Japanese Patients with Type 2 Diabetes

**DOI:** 10.1155/2014/639489

**Published:** 2014-08-03

**Authors:** Yuji Tajiri, Seiko Kawano, Saori Hirao, Tamami Oshige, Shinpei Iwata, Yasuhiro Ono, Chizuko Inada, Tomoyuki Akashi, Hideki Hayashi, Yuichi Sato, Masayuki Tojikubo, Kentaro Yamada

**Affiliations:** ^1^Division of Endocrinology and Metabolism, Department of Internal Medicine, Kurume University School of Medicine, 67 Asahi-Machi, Kurume 830-0011, Japan; ^2^Municipal Yame General Hospital, Yame 834-0034, Japan; ^3^Kouhoukai Takagi Hospital, Okawa 831-0016, Japan; ^4^Inada Clinic, Kurume 830-0039, Japan; ^5^Shimada Hospital, Ogori 838-0141, Japan; ^6^Tenjinkai Koga Hospital 21, Kurume 839-0801, Japan; ^7^Saint Mary Hospital, Kurume 830-8543, Japan; ^8^Tenjinkai Shin-Koga Hospital, Kurume 830-8577, Japan

## Abstract

*Aims*. Efficacy and safety of DPP-4 inhibitor, sitagliptin, add-on therapy to insulin were investigated in Japanese patients with type 2 diabetes. *Subjects and Methods*. Two hundred and sixteen patients (126 men, 65 ± 12 years old, BMI 24.9 ± 4.5, means ± S.D.) who had been treated by insulin alone or insulin combined with other oral hypoglycemic agents (OHAs) were recruited, and sitagliptin was added for 3 months. *Results*. HbA1c was significantly decreased after 3 months of add-on therapy as a whole (8.56 ± 1.50% to 7.88 ± 1.25%, *P* < 0.0001). Body weight did not change and insulin dosage was significantly (*P* < 0.0001) decreased for 3 months. Furthermore, day-to-day glucose variability was significantly reduced (18.3 ± 9.1 to 16.1 ± 8.1%, *P* < 0.05). In stepwise multiple regression analysis on ΔHbA1c as an outcome variable, the higher baseline HbA1c value and a preserved CPR were selected as significant predictive variables. Fifteen patients complained of mild hypoglycemia without any assistance during 3 months of sitagliptin add-on, while no severe hypoglycemic episode was reported. *Conclusions*. Add-on of sitagliptin to ongoing insulin therapy effectively reduced either HbA1c level or glucose fluctuation and could be a practical and well-tolerated alternative to treat Japanese patients with type 2 diabetes who had been inadequately controlled by insulin with or without other OHAs.

## 1. Introduction

Insulin therapy is recognized to possess the most striking and definite effect on glucose lowering in diabetic patients with the inevitable adverse effects such as hypoglycemia and weight gain. In this aspect, many previous studies have reported the efficacy and safety of combination therapy of oral hypoglycemic agents (OHAs) and insulin, indicating advantages such as the reduction of the insulin dose, the prevention of weight gain or hypoglycemia, and the improvement of glycemic control [1]. In most previous studies, however, insulin was added to ongoing OHAs, and effects of add-on therapy of OHAs to ongoing insulin therapy have seldom been discussed so far.

Sitagliptin is a selective DPP-4 inhibitor and it was approved for the treatment of type 2 diabetes mellitus (T2DM) at the end of 2009 in Japan. Many reports have demonstrated the efficacy and safety of sitagliptin as monotherapy or in combination with other OHAs, in which sitagliptin was generally well-tolerated and brought about a significant improvement of blood glucose control [2, 3]. The efficacy and safety of sitagliptin when added to ongoing insulin therapy have recently been reported in countries other than Japan [4–6]. Since the combination therapy of sitagliptin with insulin was also approved in 2011 in Japan, this add-on therapy has enthusiastically been discussed and recently some reports of Japanese patients have been published. In most studies, a definite glucose-lowering effect without any severe adverse events (SAEs) has been demonstrated in either prospective or retrospective manner [7–9]. However, one report is limited to insulin monotherapy (premixed or long acting insulin) in spite of its prospective manner [9], and the other one is limited to combination therapy with only basal insulin [7]. Furthermore, effects of add-on therapy of sitagliptin on glucose fluctuation have not been discussed in a large number of patients even though it was evaluated in a small number of patients by calculating data based on self-monitoring of blood glucose (SMBG) or continuous glucose monitoring (CGM) [7, 10].

The aim of the present study is to evaluate the effect of add-on of sitagliptin on blood glucose control or glucose fluctuation as well as the tolerability of this combination therapy in more than 200 Japanese type 2 diabetic patients treated with insulin and/or other OHA in real clinical practice.

## 2. Subjects and Methods

### 2.1. Patients

This is a multicenter retrospective observation study. Two hundred forty-one type 2 diabetic outpatients who had been treated with insulin with or without other OHAs and prescribed sitagliptin since December in 2009 were recruited in Kurume University Hospital and other satellite hospitals or clinics as candidates in the present study. The diagnosis of diabetes was confirmed according to the criteria of the Japan Diabetes Society [11] or by medical history of diabetes. Four patients showed positive antiglutamic acid decarboxylase antibody and were excluded from the study. Patients with severe liver dysfunction (AST > 100 U/L, ALT > 100 U/L, or *γ*-GTP > 100 U/L, *n* = 8), renal dysfunction (serum creatinine concentration > 133 *μ*mol/L, *n* = 9), known malignant disease (*n* = 3), or chronic inflammatory disease (e.g., rheumatoid arthritis, *n* = 1) were excluded from the study. The remaining 216 patients (126 men, 65 ± 12 years old, BMI 24.9 ± 4.5, means ± S.D.) finally participated in the present study. The study was performed in accordance with the Declaration of Helsinki and approved by the ethics committee of Kurume University Hospital.

Baseline characteristics and demographics are shown in Tables 1 and 3. One hundred and eighteen patients had been treated with OHAs in combination with insulin at the initiation of sitagliptin, including sulfonylurea (SU) in 22, biguanide (BG) in 93, *α*-glucosidase inhibitor (*α*-GI) in 22, and thiazolidinedione in 5 patients. A hundred and ninety patients were initiated by sitagliptin at the dosage of 50 mg, and remaining 26 patients were at 25 mg. Types of insulin therapy were classified to three categories as follows:

(1) twice or thrice daily injections of premixed insulin: *n* = 95;
(2) multiple daily injections composed of long acting insulin once or twice and rapid acting insulin at each meal time (MDI): *n* = 74;
(3) once daily injection of basal insulin: *n* = 47.

### 2.2. Protocol and Study Endpoints

A change of HbA1c level from baseline value 1 and 3 months after the add-on of sitagliptin is a primary outcome variable for the assessment of efficacy and is evaluated in relation to predictive variables such as initial HbA1c level, change in body weight, endogenous insulin reserve expressed by serum C-peptide level (CPR), glucose fluctuation, and types of medications including OHAs and insulin therapy. Changes in body weight and insulin dose for 3 months are the other endpoints as well. At the initiation of S, coadministered OHAs were ceased or reduced in 55 patients, and the dose of insulin was cut down in 9 patients according to the judgment of each physician. After the initiation of sitagliptin, the dose of coadministered OHAs was fixed, and the dose of insulin was titrated based on the results of SMBG to avoid hypoglycemia according to the judgment of each physician.

Glucose fluctuation is a secondary important outcome variable in this study. Parameters for the glucose fluctuation were calculated based on the results of SMBG in 115 patients (69 men, 65 ± 11 years old, BMI 25.0 ± 4.8). Patients were selected based on the frequency of measurements; that is, they had undertaken measurements of blood glucose at least 3 points from 7 points which include before and after each meal and before bedtime and at least 3 days per week. Results of SMBG for voluntary 3 days were collected before and 1, 3 months after the add-on of sitagliptin. Glucose fluctuations were expressed as coefficient of variations (CV = standard deviation/mean) of each measurement to avoid an influence of the size of absolute value. CV of blood glucoses in each day was calculated; then an average of the CVs for 3 days was found as within-day variability of blood glucose. CV of blood glucose at each time point for 3 days was calculated; then an average of the CVs at each time point was found as day-to-day variability as previously described (MODD: means of daily difference) [12].

HbA1c levels were measured by HPLC method (Arkray Inc., Kyoto) or Latex agglutination immunoassay (Kyowa Medex Co., Ltd., Tokyo) according to the equipment in each hospital. Serum CPR was measured by enzyme linked immune-sorbent assay.

### 2.3. Statistical Analysis

All tests were performed using JMP ver. 10 (SAS Institute Inc., USA) and all data were expressed as means ± S.D. The changes of HbA1c, body weight, insulin dose, and within-day and day-to-day variation of blood glucose during 3 months were evaluated by repeated measures ANOVA with Bonferroni test. The difference of HbA1c, body weight, and glucose fluctuation between before and 3 months after the initiation of sitagliptin was expressed as Δ variable and calculated by the subtraction of the value before add-on of sitagliptin from the value after 3 months. A relationship between ΔHbA1c and each predictive variable was analyzed using Student's *t*-test or one-way ANOVA for a nominal variable and Pearson's correlation coefficient for a continuous variable. For multivariate analysis on ΔHbA1c as an outcome variable, multiple stepwise regression analysis was performed. *P* values less than 0.05 were considered as statistically significant.

## 3. Results and Discussion

### 3.1. Changes in Parameters after the Initiation of Sitagliptin

Changes in each variable for 3 months are shown in Table 2. HbA1c was significantly reduced as early as 1 month after the initiation of sitagliptin and further reduced after 3 months reaching the mean reduction of 0.68% in all patients. However, no improvement was observed in 79 patients whose HbA1c levels did not decrease compared to those at baseline (8.33 ± 1.32 to 8.49 ± 1.244%, nonresponders). In spite of improvement of blood glucose level, body weight did not change significantly throughout this period. After add-on of sitagliptin, total insulin dose per day was significantly cut down to avoid hypoglycemia according to the judgment of each physician. Consequently, insulin dose could be decreased in 70 patients and insulin therapy could be ceased in 5 patients.

Regarding glucose fluctuation, day-to-day variability was significantly reduced 1 month after the add-on of sitagliptin and the reduction continued up to after 3 months, although within-day variability showed nonsignificant tendency of curtailment.

### 3.2. Parameters Which Influence the Reduction of HbA1c Level

The reduction of HbA1c value (ΔHbA1c) was evaluated in relation to each predictive variable. As shown in Table 3, among nominal variables, the combination with *α*-GI was negatively correlated and the use of premixed insulin was positively correlated to the reduction of HbA1c. Among continuous variables, the higher initial HbA1c and fasting CPR level and the increase in body weight for 3 months were significantly correlated to the reduction of HbA1c (Table 4). The changes of parameters for glucose fluctuation were not significantly related to ΔHbA1c. In multiple stepwise regression analysis on ΔHbA1c as an outcome variable, initial HbA1c level and fasting CPR level were selected as significant predictive variables (Table 5).

### 3.3. Safety

After the add-on of S, 15 patients including 9 treated with SU complained of mild hypoglycemia without any assistance, while no severe hypoglycemic episode was reported. After an appropriate cutting down of coadministered insulin dose, all those patients could avoid a recurrence of hypoglycemia thereafter. No other AEs were reported, and no patient was obliged to cease sitagliptin due to AEs.

Major outcomes of the present study were as follows. The add-on of sitagliptin for 3 months showed a significant reduction of HbA1c level in Japanese patients with T2DM whose glycemic control had not been adequate by insulin and/or OHAs without a weight gain or an occurrence of severe hypoglycemia. Sitagliptin add-on therapy could also reduce a fluctuation of blood glucose expressed as day-to-day variability. Forty-eight percent of the variation in HbA1c reduction after the add-on of sitagliptin was explained by the higher initial HbA1c level and fasting CPR level.

The add-on of sitagliptin to ongoing insulin therapy brought about the decrease in HbA1c level of around 0.7%, which is comparable to those previously reported in T2DM patients in Japan [8, 9] or in overseas countries [4, 6]. Three of them are randomized controlled studies [4, 6, 9], and the remaining one is a retrospective observation study same as the present one. Apart from the reports from overseas, one report is limited to insulin monotherapy (premixed or long acting insulin) in spite of its prospective manner [9] and the other retrospective study consists of a relative small number [8] in Japanese T2DM patients. Even though this is a retrospective one, our current study revealed, for the first time, an efficacy of sitagliptin add-on therapy to insulin in real clinical practice among a large number of patients.

Weight gain or hypoglycemia is an inevitable adverse experience in T2DM patients especially treated with SU or insulin. During the add-on therapy of sitagliptin, body weight was stable in line with a previous report [6] in which an increase of insulin dose significantly produced weight gain in spite of a stable weight observed in sitagliptin add-on therapy. Same as most reports regarding sitagliptin add-on therapy [4, 6, 8, 9], a few reports of mild hypoglycemia were observed in the present study, indicating an excellent tolerability of this combination therapy. Consequently, insulin doses were cut down in one-third of total patients and no recurrence of hypoglycemia was observed thereafter, suggesting the importance of an adjustment of insulin dose without delay referring to the results of SMBG after the add-on of sitagliptin.

To predict the efficacy of sitagliptin add-on therapy on the change of HbA1c (ΔHbA1c) is a primary interest of the present study. Initial HbA1c level was the strongest predictor in line with most previous reports [8, 13]. Otherwise, increasing of body weight, the higher serum CPR, and the use of premixed insulin positively and the combination to *α*-GI negatively contributed to the reduction of HbA1c in univariate analysis. Weight gain for 3 months might be a consequence of the blood glucose improvement. Because the principal effect of DPP-4 inhibitor is an enhancement of endogenous insulin secretion as well as an inhibition of glucagon secretion and gastric emptying [14], it is quite conceivable that the higher endogenous insulin reserve positively contributes to glucose-lowering of this drug. Although it is of interest which type of insulin therapy is more effective for glucose lowering when combined with sitagliptin, conflicting results have been reported concerning this issue so far. One observational study reported the more effectiveness with the combination of basal insulin therapy [8], whereas other two randomized control studies reported no difference among types of insulin therapy [4, 9]. In the present study, premixed insulin therapy was the most effective on the reduction of HbA1c, whereas the superiority diminished in multiple regression analysis together with weight gain and the combination to *α*-GI. A fasting serum C-peptide level was selected as a significant explanatory variable together with an initial HbA1c level after multivariate analysis, suggesting preserved endogenous insulin capacity is a clinically important factor for the prediction of the efficacy to sitagliptin add-on to ongoing insulin therapy. A recently published retrospective study which is similar to our study demonstrated that an increased total daily insulin dose and prolonged diabetes duration contribute to the attenuation of HbA1c reduction independent of baseline HbA1c level [13]. In their study, however, endogenous insulin such as CPR was not measured and it is thus plausible that those two factors may have been a reflection of the lower insulin reserve in those patients.

Our secondary important outcome is the effect of sitagliptin add-on to insulin on glucose fluctuation which has been evaluated in a small number of patients by calculating data based on self-monitoring of blood glucose (SMBG) or continuous glucose monitoring (CGM) [7, 10] so far. Although mean amplitude of glucose (MAGE) based on CGM data is a gold standard for the estimation of glucose fluctuation, it is inadequate for a large number of patients. In the present study, we calculated glucose fluctuation based on practically available SMBG data in each patient instead of CGM data. Sitagliptin add-on therapy significantly reduced day-to-day glucose variability, and this is the first report in a large number of Japanese T2DM patients. Within-day variability tended to decrease but did not reach a significant difference, probably because points of measurements are limited (4.6 ± 1.7 times per day) and most patients preferred to measure their blood glucose levels before meal rather than after meal. The change of glucose fluctuation was not related to ΔHbA1c in the present study, suggesting an effect on glucose stability is independent of that on HbA1c. Future studies are expected to confirm this issue.

There are some limitations in the present study. This is a retrospective observation study and participating physician is limited to specialist in diabetes treatment, implying the possibility of selection bias for the patients. In other word, results in the present study could reflect the actual conditions of clinical practice for diabetes treated by specialists. Because the changes of insulin dose were put into each physician's hand to avoid hypoglycemia, the efficacy and safety of the add-on therapy of sitagliptin might be different from those in physicians who are not specialists in diabetes. As mentioned above, frequency and measure point of SMBG were modestly different among patients and measurements were mainly performed before meal because they were put into each patient's hands. SMBG prior to meals, occasionally postprandially, is recommended as standards of medical care in diabetes in American Diabetes Association [15], and premeal measurements are easier and acceptable in many patients than postmeal ones.

## 4. Conclusions

From the results obtained in the present study, add-on of sitagliptin to ongoing insulin therapy could be an effective and well-tolerated alternative to treat Japanese patients with type 2 diabetes who had been inadequately controlled by insulin with or without other OHAs. Notably, this combination therapy could suppress glucose fluctuation as well as mean glucose levels. Future studies of prospective manner with a large number of patients and with more frequent postmeal SMBG data or with CGM data are warranted to confirm these findings.

## Figures and Tables

**Table 1:** Characteristics of diabetic patients.

Number of patients	216
Gender (male/female)	126/90
Age (years old)	65 ± 12
Duration of diabetes (years)	15.8 ± 9.1
BMI	24.9 ± 4.5
Initial HbA1c (%)	8.56 ± 1.50
Initial dose of insulin (U/day)	29.0 ± 18.6
Fasting serum CPR (nmol/L)^a^	0.47 ± 0.37
Postprandial serum CPR (nmol/L)^b^	0.79 ± 0.44
Within-day glucose variability (%)^c^	23.8 ± 10.5
Day-to-day glucose variability (%)^c^	18.6 ± 9.1

Abbreviations are shown in text. Data are expressed as means ± S.D. or numbers of patients. ^a^ *n* = 102, ^b^ *n* = 96, and ^c^ *n* = 115.

**Table 2:** Changes in parameters for 3 months.

	Before	1 month	3 months
HbA1c (%)	8.56 ± 1.5	8.13 ± 1.26^**^	7.88 ± 1.25^∗∗†^
Body weight (kg)	63.3 ± 12.9	63.2 ± 12.9	63.5 ± 12.9
Insulin dose (U/day)	29 ± 18.6	27.7 ± 18.8^**^	27.4 ± 18.9^∗∗#^
Within-day glucose variability (%)^a^	24 ± 10.7	22.7 ± 10.3	22.6 ± 11.4
Day-to-day glucose variability (%)^a^	18.3 ± 9.1	16.8 ± 8.4^*^	16.1 ± 8.1^*^

Abbreviations are shown in text. Repeated measures ANOVA with Bonferroni test.

Data are expressed as means ± S.D. ^a^ *n* = 115.

^*^ *P* < 0.05, ^**^ *P* < 0.001 versus before the initiation of sitagliptin.

^#^ *P* < 0.05, ^†^ *P* < 0.001 versus 1 month after the initiation of sitagliptin.

**Table 3:** Relationship between ΔHbA1c and each nominal variable.

Predictive variable	*n*	ΔHbA1c (%)	*P* value
Gender			
Male	124	−0.67 ± 1.28	N.S.
Female	92	−0.66 ± 0.90	

Combined with other OHAs			
None	98	−0.67 ± 0.98	N.S.
+	118	−0.66 ± 1.25	

Combined with SU			
None	194	−0.68 ± 1.16	N.S.
+	22	−0.51 ± 0.81	

Combined with BG			
None	123	−0.65 ± 1.08	N.S.
+	93	−0.69 ± 1.20	

Combined with *α*-GI			
None	194	−0.72 ± 1.10	0.033
+	22	−0.10 ± 1.25	

Insulin therapy			
Premixed	95	−0.96 ± 1.31	0.002
MDI	74	−0.48 ± 0.98	
Basal insulin	47	−0.35 ± 0.78	

Abbreviations are shown in text. Student's *t*-test or one-way ANOVA.

Δ = (value after 3 months) − (value before add-on of sitagliptin).

Premixed: twice or thrice daily injections of premixed insulin. MDI: multiple daily injections composed of long acting insulin once or twice and rapid acting insulin at each meal time. N.S.: not significant.

**Table 4:** Relationship between ΔHbA1c and each continuous variable.

Predictive variable	*r* value	*P* value
Age (years old)	0.064	N.S.
Duration of diabetes (years)	0.102	N.S.
Initial BMI	−0.039	N.S.
Initial HbA1c (%)	−0.577	<0.0001
ΔBody weight (kg)	−0.196	0.005
Dose of insulin (U/day)	−0.008	N.S.
Fasting serum CPR (nmol/L)^a^	−0.222	0.026
Postprandial serum CPR (nmol/L)^b^	−0.08	N.S.
ΔWithin-day glucose variability (%)^c^	0.102	N.S.
ΔDay-to-day glucose variability (%)^c^	0.107	N.S.

Abbreviations are shown in text. Pearson's correlation coefficient.

Δ = (value after 3 months) − (value before add-on of sitagliptin).

^a^ *n* = 102, ^b^ *n* = 96, and ^c^ *n* = 115. N.S.: not significant.

**Table 5:** Relationship between ΔHbA1c and each variable.

Predictive variable	Partial regression coefficient	Standard error	*t* value	*P* value
Initial HbA1c (%)	−0.361	0.05	−7.22	<0.0001
Fasting serum CPR (ng/mL)	−0.211	0.084	−2.5	0.015
Insulin therapy (premixed versus basal insulin)	−0.184	0.139	−1.32	0.189
Insulin therapy (premixed versus MDI)	−0.087	0.121	−0.72	0.473
Combined with *α*-GI	−0.065	0.15	−0.43	0.666
ΔBody weight (kg)	0.009	0.041	0.22	0.824

Abbreviations are shown in text. Multiple regression analysis. *r* ^2^ = 0.48 in this model.

Δ = (value after 3 months) − (value before add-on of sitagliptin).

Abbreviations for insulin regimens are shown in footnote of Table 3.
